# Can duodenal resection be a valid alternative to pancreatoduodenectomy for nonampullary duodenal tumors?

**DOI:** 10.1590/0102-672020260000036e1965

**Published:** 2026-08-28

**Authors:** Teresa PERRA, Alberto PORCU

**Affiliations:** 1 Azienda Ospedaliero Universitaria di Sassari, Department of General Surgery – Sassari, Italy.

**Keywords:** General Surgery., Duodenal Neoplasms, Duodenal Diseases, Pancreaticoduodenectomy, Cirurgia Geral, Neoplasias Duodenais, Duodenopatias, Pancreaticoduodenectomia

## Abstract

**Background:**

Pancreatoduodenectomy is still the most common surgical treatment for patients with duodenal tumors. However, in selected cases where duodenal resection is technically feasible, it could represent a valid alternative, reducing the risk of postoperative complications, being a less invasive procedure.

**Aims:**

The objective of this study was to evaluate the results of this procedure at our institution.

**Methods:**

We collected data from all patients undergoing duodenal resection for oncologic disease between January 2020 and June 2025 at our institution. After a multidisciplinary evaluation, duodenal resection was indicated when the distance between the duodenal tumor and papilla of Vater measured endoscopically was at least 2 cm, and the tumor was operable with radical intent.

**Results:**

Eight patients were treated with this procedure. No major postoperative complications were observed. Two patients had postoperative nausea and vomiting, which resolved with antiemetics. Average hospital stay was five days. There has been no recurrence or death to date.

**Conclusions:**

Duodenal resection seems a valid alternative to pancreatoduodenectomy for the treatment of duodenal tumors in selected cases. It reduces the risk of postoperative complications, eliminating the risk of complications specific to pancreatoduodenectomy, such as pancreatic and biliary fistulas. It is a less invasive and shorter procedure, less severely compromises the patients general condition, and reduces hospital stay. Further studies are needed to confirm these results and draw generalizable conclusions.

## ARTICLE HIGHLIGHTS

Duodenal resection seems a valid alternative to pancreatoduodenectomy for the treatment of non-ampullary duodenal tumors in selected cases.

It reduces the risk of postoperative complications.

It eliminates the risk of complications specific to pancreatoduodenectomy, such as pancreatic and biliary fistulas.

It is a less invasive and shorter procedure, less severely compromises the patient’s general condition, and reduces hospital stay.

## Summary

**CENTRAL MESSAGE:**

Pancreatoduodenectomy is still the standard procedure in the surgical treatment of patients with duodenal tumors. It is a complex and technically challenging procedure that can develop severe complications. Pancreatic and biliary fistulas are common and serious complications that are specific of this surgery. In selected cases where duodenal resection is technically feasible, it could represent a valid alternative to pancreatoduodenectomy, reducing the risk of postoperative complications, being a less invasive procedure.

**PERSPECTIVES:**

Duodenal resection seems a valid alternative to pancreatoduodenectomy for the treatment of duodenal tumors in selected cases. It reduces the risk of postoperative complications, eliminating the risk of complications specific to pancreatoduodenectomy, such as pancreatic and biliary fistulas. It is a less invasive and shorter procedure, less severely compromises the patient’s general condition, and reduces hospital stay. Further studies are needed to confirm these results and draw generalizable conclusions.

## VISUAL ABSTRACT

**Figure:**
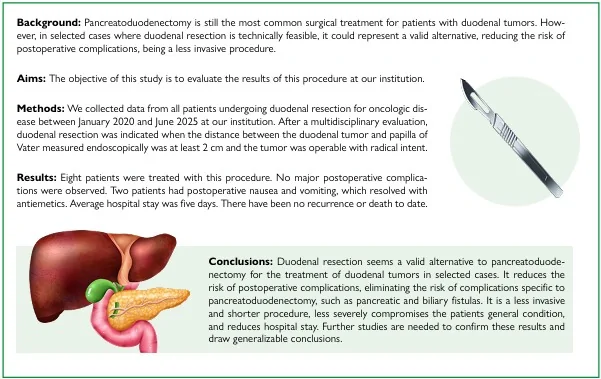

## RESUMO

**Racional:**

A pancreatoduodenectomia ainda é o tratamento cirúrgico mais comum para pacientes com tumores duodenais. No entanto, em casos selecionados nos quais a ressecção duodenal é tecnicamente viável, ela pode representar uma alternativa válida, reduzindo o risco de complicações pós-operatórias, por ser um procedimento menos invasivo.

**Objetivo:**

O objetivo deste estudo é avaliar os resultados deste procedimento em nossa instituição.

**Métodos:**

Coletamos dados de todos os pacientes submetidos à ressecção duodenal por doença oncológica entre janeiro de 2020 e junho de 2025 em nossa instituição. Após avaliação multidisciplinar, a ressecção duodenal foi indicada quando a distância entre o tumor duodenal e a papila de Vater, medida endoscopicamente, era de pelo menos 2 cm e o tumor era operável com intenção curativa.

**Resultados:**

Oito pacientes foram tratados com este procedimento. Não foram observadas complicações pós-operatórias importantes. Dois pacientes apresentaram náuseas e vômitos pós-operatórios, que foram resolvidos com antieméticos. A média de internação hospitalar foi de cinco dias. Não houve recidivas ou óbitos até o momento.

**Conclusões:**

A ressecção duodenal parece ser uma alternativa válida à pancreatoduodenectomia para o tratamento de tumores duodenais em casos selecionados. Ela reduz o risco de complicações pós-operatórias, eliminando o risco de complicações específicas da pancreatoduodenectomia, como fístulas pancreáticas e biliares. É um procedimento menos invasivo e mais curto, que compromete menos o estado geral do paciente e reduz o tempo de internação hospitalar. Mais estudos são necessários para confirmar esses resultados e tirar conclusões generalizáveis.

## INTRODUCTION

 Pancreatoduodenectomy is still the standard procedure in the surgical treatment of patients with duodenal tumors. It is a complex and technically challenging procedure that can develop severe complications. Pancreatic and biliary fistulas are common and serious complications that are specific of this surgery. 

 In selected cases where duodenal resection is technically feasible, it could represent a valid alternative to pancreatoduodenectomy, reducing the risk of postoperative complications, being a less invasive procedure. 

 Oncological outcomes should be carefully analyzed in order to confirm this surgical procedure as a gold standard, defining the precise indications for this kind of surgery. 

 Some authors have proposed duodenal resection in the surgical treatment of patients with duodenal tumor. It seems a promising technique, but there are still few studies in scientific literature, and most of them have small sample size. Iwasaki et al.^8^ showed a single center experience and a systematic review of the literature about the surgical treatment of neuroendocrine tumors in the second portion of the duodenum. They underlined the importance of clarifying the prognostic impact of lymph node dissection^8^. Cloyd et al.^5^ performed a literature review about advances in diagnosis and surgical management of duodenal adenocarcinoma. It is an uncommon cancer, and previous studies have generally combined it with other periampullary or small bowel cancers, limiting the possibility to draw generalizable conclusions. They conclude that both pancreatoduodenectomy and segmental duodenal resection are acceptable surgical options if margins are negative and lymphadenectomy is adequate. Another important point to consider is the possibility of multi-modality treatment for patients at high risk of recurrence, such as adjuvant chemotherapy and radiation^5^. Li et al.^9^ performed a systematic review about the outcomes of surgical resection for primary duodenal adenocarcinoma. The evidence about the kind of resection required for this kind of cancer is still debated. From the oncological point of view, it seems that pancreas-sparing duodenectomy should be considered if tumor invasion is confined in the duodenal wall, especially for distal duodenal tumors^3,9^. 

 The review of the literature by Hashimoto et al.^7^ showed the studies of limited resection of the duodenum for nonampullary duodenal tumors. They underlined how the surgical management of duodenal diseases could be challenging, due to their retroperitoneal position and because supply is shared with the pancreas. Endoscopic resection and limited duodenal resection were described. They concluded that limited duodenal resections were safe and feasible procedures in case of superficial or small nonampullary duodenal lesions. Laparoscopic surgery could be an option. However, the optimal surgical management is still controversial^7^. 

 Bourke et al.^4^ performed an expert review about clinical practice update for nonampullary duodenal lesions. As they wrote, although most nonampullary duodenal polyps are benign, adenomas are estimated to be about 10–20% of these lesions, and most international guidelines recommend that all duodenal adenomas should be considered for endoscopic resection^4^. If it is not possible, surgical resection is indicated. 

 The aim of this study is to evaluate the safety, technical feasibility and main outcomes of duodenal resection in the surgical treatment of patients with duodenal tumor at our institution. 

## METHODS

 We performed a Strengthening the Reporting of Observational Studies in Epidemiology (STROBE)-conformed^14^ single institution retrospective study. We collected and analyzed data from all patients undergoing duodenal resection for oncological disease from January 2020 to June 2025 at our institution. 

### Inclusion and exclusion criteria

 Patients were included if they underwent duodenal resection for oncological disease. Patients were excluded if they underwent pancreatoduodenectomy, or presented other tumors or diseases that could influence prognostic outcomes. 

### Preoperative management

 All patients underwent preoperative evaluation with wholebody CT and EUS-FNB. After a multidisciplinary evaluation, duodenal resection was indicated when the distance between the duodenal tumor and papilla of Vater measured endoscopically was at least 2 cm and the tumor was operable with radical intent. All patients underwent oncologic evaluation once the histological report of the surgical specimen was available. 

### Surgical operation

 The surgical operation began with an extensive mobilization of the duodenum (Kocher’s manoeuvre). Then the mesentery of the duodenojejunal flexure was ligated and divided, and the upper jejunum transected. The duodenojejunal specimen was passed under the superior mesenteric vessels into the supracolic compartment. The third portion of the duodenum was carefully separated from the head of the pancreas. Then the duodenum was divided 2–3 cm under the major duodenal papilla. Finally, an end-to-end duodenojejunal anastomosis was performed. In particular, a continuous suture was performed on the posterior wall, while an interrupted suture was made on the anterior wall. 

### Data collected

 The following information was collected for each patient: age, sex, comorbidities, type and characteristics of the tumor, type of surgery, oncological staging, possible neoadjuvant and/or adjuvant chemotherapy and/or radiotherapy, length of hospital stay, postoperative complications, survival, and local and/or distant recurrence. 

 The study was conducted in accordance with the Declaration of Helsinki, and approved by the Institutional Review Board of AOU Sassari (Number DEL/2022/320). 

### Statistical analysis

 Qualitative variables are presented as frequencies and percentages. Quantitative variables are presented as means and standard deviation or medians and ranges, when necessary (the Shapiro-Wilk normality test was used to verify the normality of the distribution of the variables). Statistical analysis was performed using Microsoft Excel 2016 (Microsoft, Redmond, WA, USA) and R (R Foundation for Statistical Computing, Vienna, Austria) statistical software. 

## RESULTS

 The present study included eight patients who underwent duodenal resection for oncological disease. Patients’ characteristics are presented in Table 1. 

**Table 1:** Patients’ characteristics.

Variables			N n=8
Age (years, mean±SD)			65.5 (11.55)
BMI (kg/m^2^, mean±SD)			25.13 (1.46)
Sex			
	Male (n (%))		6 (75)
	Female (n (%))		2 (25)
	CCI (mean ± SD)		4.13 (0.83)
Pathological type			
	Benign tumor (n (%))		3 (37.5)
		GIST (n (%))	2 (25)
		NET (n (%))	1 (12.5)
	Adenocarcinoma (n (%))		2 (25)

BMI: body mass index; CCI: Charlson Comorbidity Index; GIST: gastrointestinal stromal tumors; NET: neuroendocrine tumor; SD: standard deviation.

 Three of them were affected by benign tumors that could not be treated with an endoscopic approach. Two patients were diagnosed with primary non-metastatic duodenal gastrointestinal stromal tumors (GIST). One patient was diagnosed with a duodenal neuroendocrine tumor (NET). Two other patients presented duodenal adenocarcinoma and, after a multidisciplinary evaluation and a careful discussion on risks and benefits, considering the initial state of the tumor, the distance of the tumor from the papilla of Vater >2 cm measured endoscopically, and the absence of local and distance invasion, they agreed to undergo this procedure with close clinical follow-up. 

 The outcomes are presented in Table 2. No major postoperative complications were observed. Two patients had postoperative nausea and vomiting, which was resolved with antiemetics. The average hospital stay was five days. There has been no recurrence or death to date. 

**Table 2:** Patients’ outcomes.

Outcomes	N n=8
PONV (n (%))	2 (25)
Complications (Clavien-Dindo ≥3 (n (%))	0 (0)
30-day mortality (n (%))	0 (0)
1-year recurrence (n (%))	0 (0)
Hospital stay (days, mean±SD)	5.13 (0.99)

PONV: postoperative nausea and vomiting; SD: standard deviation.

## DISCUSSION

 A wide range of diseases with a different grade of malignancy can represent a surgical indication for limited duodenal resection. Currently, the most common indications are duodenal adenomas, gastrointestinal stromal tumors (GISTs), neuroendocrine tumors and early duodenal carcinomas. 

 Limited resection is indicated in case of any benign neoplasm or ulceration of the distal duodenum. It includes Crohn’s disease if medical treatment fails^11^. If endoscopic resection of duodenal adenomas is not feasible or has failed, surgical resection is indicated. GISTs have a low propensity to invade locally and metastasize when they arise in the duodenum, so they can ideally be treated with limited resections^6^. 

 For benign lesions and tumors without local and distance invasion, duodenal resection surely represents a valid option to pancreatoduodenectomy. In case of malignant tumors, like adenocarcinoma, precise criteria should be created in order to reduce the risk of recurrence. A distance between the tumor and papilla of Vater of at least 2 cm measured endoscopically seems a safe and useful cut-off that can be used as indication for surgery. Further studies are needed to confirm our findings, and for this cut-off to be used in clinical practice as a surgical indication. 

 Duodenal resection seems to provide many advantages. It reduces the risk of postoperative complications, eliminating the risk of pancreatic and biliary fistulas that are complications specific to pancreatoduodenectomy. It is a shorter and less invasive surgical procedure that less severely compromises the patient’s general condition. Hospital stay is also shorter. 

 As some authors showed, limited duodenal resection for nonampullary lesions can avoid morbidity related to pancreatoduodenectomy and preserve pancreatic function^1,2,7,11-13^. An important point to consider is that almost no local recurrence has been reported after limited duodenal resection^1,7^. 

 Recently, endoscopic resection and laparoscopic surgery have been reported^2,13^. Although long-term outcomes have not been studied enough, these procedures are less invasive and have shown safe and feasible short-term outcomes^1,2,7,12,13^. 

 Pancreas-sparing duodenal resection is a technically demanding surgical procedure that should be performed by a skilled surgeon. It eliminates the need for pancreatic resection and is associated with good absorptive capacity, weight gain, and better quality of life. It can reduce the risk of subsequent malignancy, although long-term surveillance is still required^10^. 

 Duodenal resection seems a promising technique in case of benign lesions or duodenal GIST that cannot be removed with an endoscopic approach, but also in case of patients affected by duodenal NET or adenocarcinoma. In these cases, we recommend that the distance of the tumor from the papilla of Vater be at least 2 cm, and the absence of local and distance invasion is a mandatory criteria. 

 The risk of recurrence should be carefully evaluated. A multidisciplinary team should always evaluate any case before performing a duodenal resection. A careful discussion of risks and benefits should always be performed with the patient before indicating this surgical procedure. 

 In the last years, there have been many advancements in chemotherapy and radiotherapy. Surgical indications could be wider in the future. 

 We acknowledge the limitations of the present study. It is a retrospective study with a small sample size. The indications for pancreas-preserving duodenal resection can be very different. Our results should be confirmed by larger prospective studies and a randomized clinical trial, if possible, to draw generalizable conclusions. 

## CONCLUSIONS

 Duodenal resection seems a valid alternative to pancreatoduodenectomy for the treatment of duodenal tumors in selected cases. It reduces the risk of postoperative complications, eliminating the risk of complications specific to pancreatoduodenectomy, such as pancreatic and biliary fistulas. It is a less invasive and shorter procedure, it compromises the patient’s general condition less severely, and reduces hospital stay. Further studies are needed to confirm these results and draw generalizable conclusions. 

## Data Availability

The datasets generated and/or analyzed during the current study are available from the corresponding author upon reasonable request. The information regarding the investigation, methodology and data analysis of the article is archived under the responsibility of the authors.
